# Pressure Infusion Cuff and Blood Warmer during Massive Transfusion: An Experimental Study About Hemolysis and Hypothermia

**DOI:** 10.1371/journal.pone.0163429

**Published:** 2016-10-06

**Authors:** Thomas G. Poder, Denise Pruneau, Josée Dorval, Louis Thibault, Jean-François Fisette, Suzanne K. Bédard, Annie Jacques, Patrice Beauregard

**Affiliations:** 1 UETMIS, CIUSSS de l’Estrie—CHUS, Sherbrooke, Québec, Canada, J1G 2E8; 2 CRCHUS, CIUSSS de l’Estrie—CHUS, Sherbrooke, Québec, Canada, J1H 5N4; 3 Blood Bank, CIUSSS de l’Estrie—CHUS, Sherbrooke, Québec, Canada, J1H 5N4; 4 Research and Development division, Héma-Québec, Québec, Québec, Canada, G1V 5C3; 5 Hematology-Oncology division, CIUSSS de l’Estrie—CHUS, Sherbrooke, Québec, Canada, J1H 5N4; Fraunhofer Research Institution of Marine Biotechnology, GERMANY

## Abstract

**Background:**

Blood warmers were developed to reduce the risk of hypothermia associated with the infusion of cold blood products. During massive transfusion, these devices are used with compression sleeve, which induce a major stress to red blood cells. In this setting, the combination of blood warmer and compression sleeve could generate hemolysis and harm the patient. We conducted this study to compare the impact of different pressure rates on the hemolysis of packed red blood cells and on the outlet temperature when a blood warmer set at 41.5°C is used.

**Methods:**

Pressure rates tested were 150 and 300 mmHg. Ten packed red blood cells units were provided by Héma-Québec and each unit was sequentially tested.

**Results:**

We found no increase in hemolysis either at 150 or 300 mmHg. By cons, we found that the blood warmer was not effective at warming the red blood cells at the specified temperature. At 150 mmHg, the outlet temperature reached 37.1°C and at 300 mmHg, the temperature was 33.7°C.

**Conclusion:**

To use a blood warmer set at 41.5°C in conjunction with a compression sleeve at 150 or 300 mmHg does not generate hemolysis. At 300 mmHg a blood warmer set at 41.5°C does not totally avoid a risk of hypothermia.

## Background

In the last four decades, transfusion practices in trauma and other clinical situations (e.g. obstetrics, surgery, intensive care) have changed significantly. Ramakrishnan and Cattamanchi [1] present an overview of these changes and highlight that hypothermia is still a major problem due to hypovolemia and massive transfusion of cold blood products. A massive transfusion is historically defined as a patient being transfused with ten or more packed red blood cells (RBCs) within first 24 hours [2]. With the advent of more rapid therapy, alternative definitions have been used, such as a transfusion of three or four units over one hour [3–5]. Because blood products are stored at temperatures between 1 and 6°C [6], their transfusion in human body has the potential to contribute to hypothermia, which may affect the metabolism and can cause an arrhythmia or a cardiac arrest [7–9]. To overcome this problem, the use of blood warmer became more widespread [10–11]. However, the warming of blood products is associated with a risk of hemolysis [8,11]. Most common consequences in patients transfused with hemolyzed blood products are fever, kidney failure, hypotension and disseminated intravascular coagulation [12–14]. These issues raised the question of whether the mechanical fragility of red cell was increased by warming and generate hemolysis [10]. Actually, indications for warming blood products are defined by the American Association of Blood Banks in their "Guidelines for the use of blood warming devices" [15]. However, these guidelines provide no indication as regard to the optimal temperature warming and the pressure that can be exerted on blood products. It just mentioned that the heating to a temperature above 37°C may cause hemolysis. Actually, it is considered that the patient safety could be compromised with a percentage of hemolysis higher than 0.8% or 1% [16,17]. Considering that the fragility of the RBC membrane increase with temperature heating [18] and that any additional pressure exerted on it have the potential to generate hemolysis [19], it is important to examine whether to heat a blood product at a high temperature during massive transfusion with a compression sleeve can generate hemolysis at a percentage higher than those internationally admitted.

Although out of the scope of our paper, we should also mention that other factors than hypovolemia and massive transfusion can contribute to hypothermia, namely, in the pre-hospital phase, the severity of injury (e.g. head injury, spinal cord injury, shock), extremes of age, wet clothing, general anesthesia and pre-hospital intubation [20]. In the hospital phase exposure, size of surgery, cold intravenous fluids, burns, general, epidural or spinal anesthesia contribute to hypothermia [20]. In this setting, different warming methods have been used to control hypothermia: removing wet clothing, warming blankets use, heated air mattress, hot packs, fluid warmers, body cavity lavage, humidified gases and continuous arteriovenous rewarming [20].

Actually, few studies have tested the effect of high pressure in conjunction with RBCs warming, and no one was with a blood warmer set at a temperature higher than 39.8°C. In these studies, a manual pressure pump or a compression sleeve/pressure infusor were used. These devices exert a positive pressure on the packed blood product which generally flows through a tubing that is inserted in the blood warmer. However, in some studies, the blood product was pre-warmed before the pressure tests [10,21]. The study by Du Plessis and al. [10] showed that transfusion of pre-warmed blood (32–36°C) generated significantly much less hemolysis than transfusion of cold blood at 80–120mmHg. However, no difference was observed when blood at ambient temperature (18–20°C) was used. At a pressure rate of 300 mmHg, the difference in hemolysis with blood at ambient temperature and pre-warmed blood was significantly in favor of this last one. This study, however, did not provide a direct comparison between different pressure rates with the same blood product. Later, Linko [21] indicated no hemolysis increase in pre-warmed blood at 37°C for different pressure rates, but did not clearly indicate these rates. From their side, Mateer et al. [19] investigated the effect of pressure at 300 mmHg and 600 mmHg on various catheters with a blood warmer set at 36.7°C. They found a small increase (about 10%) in free hemoglobin between the two pressure tested for outdated blood, but it was not statistically significant. As regard to the study by Pappas et al. [22], they indicated no statistically significant increase in plasma hemoglobin at 300 mmHg with blood warmers set up to 39.8°C as compared to the baseline levels (i.e. the measure before the test). Kim et al. [23] also did not show a significant increase in the hemolysis percentage at 37°C with a pressure of 300 mmHg as compared to the baseline level. Finally, Kim et al. [24], with a blood warmer set at 39°C, showed that outdated packed RBCs transfused at 300 mmHg did not present more hemolysis than the baseline level.

In this study, we used a blood warmer with countercurrent heat exchange set at 41.5°C and investigated how the pressure exerted on the blood product with a compression sleeve had an effect on the hemolysis level. Two pressure levels were tested: 150 and 300 mmHg. The main objective of this study was to determine if the use of a blood warmer set at 41.5°C with a compression sleeve is a safe procedure in terms of induced hemolysis and if there is a difference as regard to the pressure used. Then, we measured the outlet temperatures of products warmed to investigate a possible risk of hypothermia for the patient (i.e. temperature below 35°C).

## Materials and Methods

**Fig 1:**
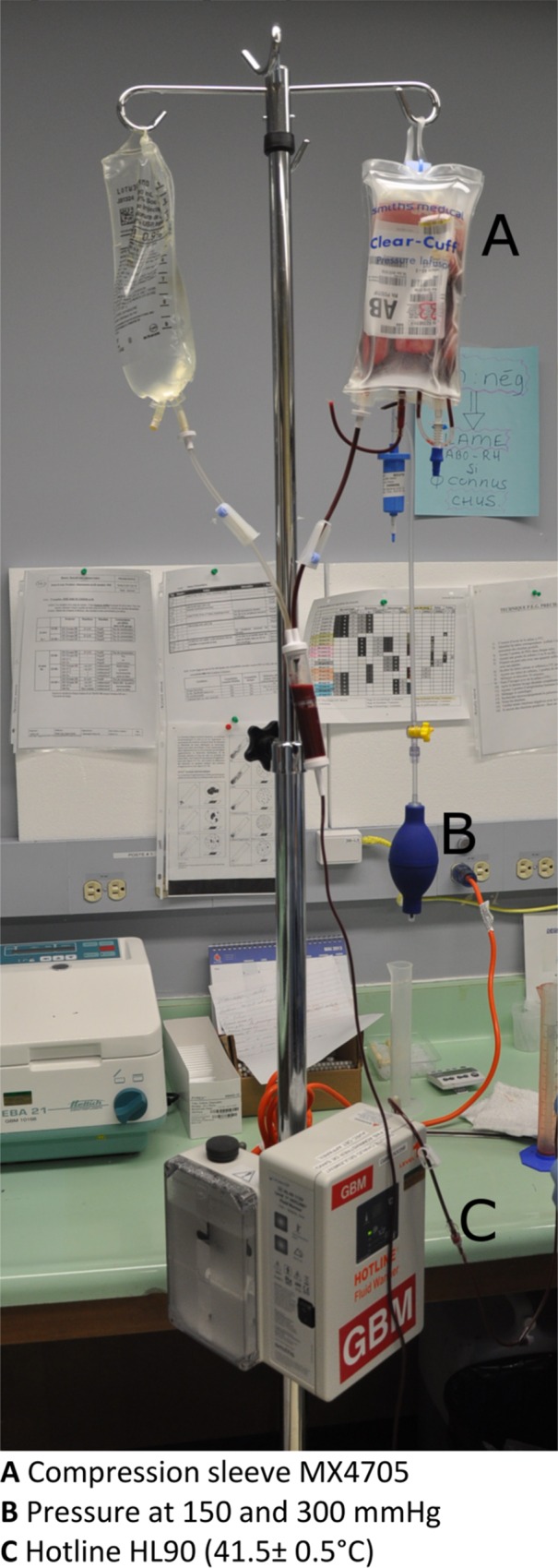
Mounting and procedure.

The study was conducted using a prospective comparative design. Each red blood cell unit was tested sequentially at 150 and 300 mmHg with a compression sleeve. The compression sleeve was a MX4705 Smiths Medical Clear-Cuff. The blood warmer we used was the Hotline® HL-90 produced by Smith Medical. With the Hotline® HL-90, the tube is inserted within the blood warmer and the wall of the tube is warmed at 41.5°C ± 0.5°C over a length of 20 centimeters. Fig 1 illustrates the mounting and general procedure used.

Ten RBC units type AB positive from 36 to 42 days were provided by Héma-Québec. These units were ready to use for transfusion. For each donor previously recruited by Héma-Québec, blood was collected with 63 mL of double-citrate phosphate dextrose (CP2D). RBC units were all prepared by adding 100 mL of adenine-saline (AS3). The average volume of the 10 RBC units was 305 ml (+/- 8 ml).

The primary outcome in this study was the level of hemolysis as a percentage. Measurement of this outcome was performed on blood sample collected before and after the passage in the blood warmer. More specifically, samples were collected just after the complete inversions of the packed RBC and then at the outlet of the tube inserted in the blood warmer. The level of free hemoglobin in g/dL was also measured as an indicator of hemolysis. Finally, we measured the temperature of RBCs at the outlet of the tube.

To perform hemolysis measurement, RBCs’ samples of 13 mL were collected. An aliquot of 3 mL from each sample was taken to perform a complete blood count (CBC). The remaining 10 mL was centrifuged at 5000 rpm for ten minutes and the supernatant transferred to a tube "free Hb". A small proportion of the supernatant (1.5 mL) was again centrifuged at 5000 rpm x 7 minutes and the resulting supernatant was filtered through a 0.22 μM filter prior to measure the free hemoglobin (FHb) level. FHb levels were measuring using a photometer HemoCue Plasma/Low HB (HemoCue, Angelholm, Sweden). To calculate the percentage of hemolysis we used the following equation: ([FHb] / [Total Hb]) X (100—Htc) where Htc is the hematocrit rate. Temperatures measured at the outlet of the tube inserted in the blood warmer were performed with a Fluke 52 Series II thermometer (+/- 0.3°C) while RBCs flowed.

### Statistical analysis

To detect a statistically significant difference between an initial level of 0.25% hemolysis and a final level of 0.8% with a standard error of 0.4, a power of 80% and significance at 95%, a minimum of 9 observations per group was required. A Shapiro-Wilk test was performed to test for distribution normality and Wilcoxon signed-rank tests were done. Data was compiled on MS Excel charts and transferred to R statistical software for analysis. A significant result was set at 95%.

### Ethical considerations

The study was approved by the Ethics Research Committee of the University Hospital of Sherbrooke (CHUS) and by the legal department of Héma-Québec. No consent from patients was needed for this study.

## Results

Tests on 10 RBC units generated a total of 29 samples for the analysis of hemolysis (10 samples before the warming with compression sleeve and 19 after). One sample collected at the outlet of a test at 150 mmHg was lost during the procedure. Tests were carried out in June 2013.

### Hemolysis

**Fig 2:**
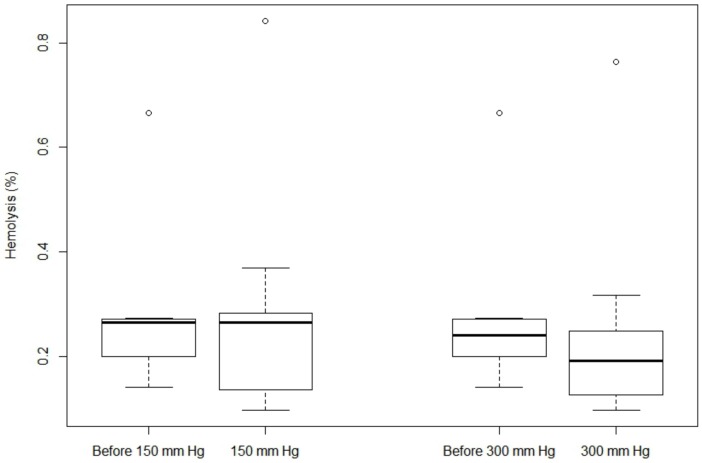
Box-plot of hemolysis (%) according to the pressure exerted.

**Fig 3:**
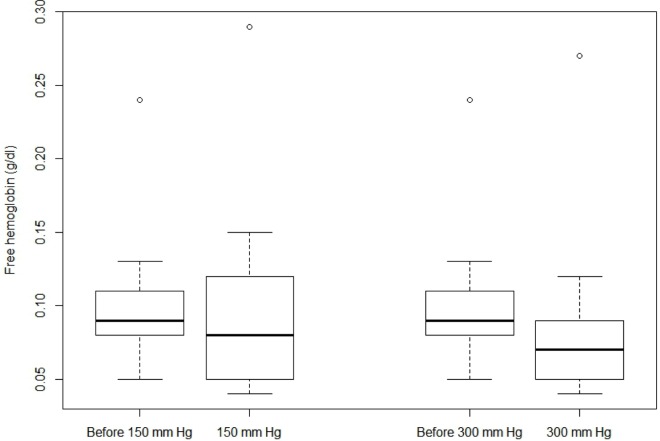
Box-plot of hemolysis (FHb in g/dl) according to the pressure exerted.

The variables of hemolysis and temperature collected did not follow a normal distribution. As a consequence, a Wilcoxson signed-rank test was used and values given are for the median. Whether it is for the percent of hemolysis or the FHb, results in Figs 2 and 3 indicate no statistically significant difference between a compression sleeve at 150 mmHg and 300 mmHg when blood was warmed at 41.5°C. Moreover, when samples before compression were compared with sample after compression (150 mm Hg or 300 mm Hg), no statistical difference was observed. Exact values are given in S1 Table.

### Temperature

**Fig 4:**
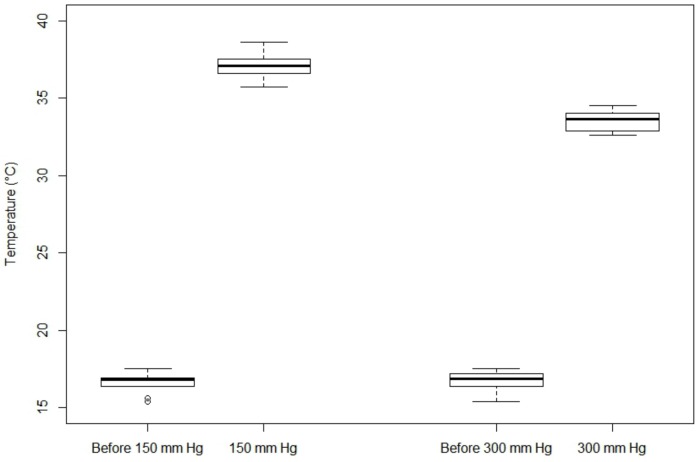
Boxplot of temperature according to the pressure exerted.

To ensure that the blood warmer effectively warm the RBCs, the temperature of RBCs was taken at the outlet of the tube (results are given in Fig 4). The rapid flow rates associated with a pressure of 150 mmHg or 300 mmHg led the blood product to a short time exposure along the 20 centimetres length of tubing in contact with the heat source. The duration of exposure to heating is thus too short to allow RBCs to achieve the desired temperature. Consequently, at a pressure of 150 and 300 mmHg, RBCs temperature reached respectively 37.1°C and 33.65°C, while the blood warmer was set at 41.5°C. Exact temperatures are given in S2 Table.

## Discussion

Along with coagulopathy and acidosis, hypothermia is a component of the “lethal triad of trauma” [25]. In his study, Spinella [26] showed that warming fresh whole blood could increase the survival rates of soldiers. However, warming whole blood or RBCs is not without risk. Indeed, warming RBCs during a transfusion can have negative consequences for the patient due to a risk of hemolysis [8,12,27]. A recent meta-analysis indicated that warming a blood product up to 45–46°C generates clinically negligible hemolysis [11]. However, this meta-analysis and other studies indicated that blood warming weakens RBCs and that excessive pressure exerted on RBCs has the potential to generate a significant hemolysis [11,18]. In our study, we tested whether the use of a blood warmer set at 41.5°C in combination with a compression sleeve induces hemolysis.

As compared to previous studies on this topic, our study is the only one that used a warming temperature higher than 39.8°C, which is important considering that the majority of blood warmers actually sold by manufacturers can be set up to 41–43°C. In addition, with the study by Pappas et al. [22], our study is only one of two which did not use outdated or improper blood product for transfusion. This is particularly important in terms of clinical validity, because RBCs used were not already weakened. An additional strength of our study is that RBCs used for comparing the two pressure levels came from the same RBC unit (i.e. the unit was tested sequentially), thus avoiding a selection bias. Our study is however limited by the fact that we did not test the effect of the compression sleeve on the level of hemolysis using an inactivated blood warmer. This limit is due to the fact that our RBC units had insufficient volume to perform these additional tests. Nevertheless, the main purpose of our study was to determine the existence of a statistically significant difference in the level of hemolysis between two high pressure levels with a blood warmer set at 41.5°C. Despite some difference with previous studies published, we found that the use of a blood warmer with high pressure is a safe practice as regard to hemolysis, even at a warming temperature of 41.5°C.

As regard to the risk of hypothermia induced by the use of a blood warmer with high pressure, our study indicated that at 150 mmHg, this risk is null (i.e. a temperature at 37.1°C). However, at 300 mmHg this risk is more important with a median temperature at the outlet of the tube that reach only 33.7°C with a maximum at 34.5°C and a minimum at 32.6°C. This drop in temperature can be compared to those observed in the studies of Mateer et al. [19] and Kim et al. [24]. In the study of Mateer et al. [19], the temperature reached 27.5 and 25.3°C with a pressure of 300 and 600 mmHg whereas the blood warmer was set at 36.7°C. The study by Kim et al. [24] showed better results in term of temperature with outlet temperature at 38.1°C for a blood warmer set at 39°C and a pressure at 300mmHg. One major difference between these two studies is the mechanism of the two blood warmers and the temperature of the blood product before heating. In the study of Mateer et al. [19], a dry heat blood warmer was used (Fenwal Model BW-5) whereas in the study of Kim et al. [24] a warming plate (ThermoSens) was used. More importantly, the inlet temperatures were 13 and 23.2°C, respectively. In contrast, in our study, we used cold RBC units (i.e. 4°C) that tempered at ambient temperature (i.e. 23–24°C) and reached 16.7°C before heating with a blood warmer set at 41.5°C. Indirect comparisons may thus indicate that the higher is the temperature at the inlet, the higher the blood warmer will perform. However, in clinical practice, it is not appropriate to wait more than 30 minutes before performing the transfusion and at 30 minutes the temperature of a packed RBC is close to 13°C. Consequently, results provided by our study are seemingly closer to clinical reality than those obtained by Kim et al. [24].

A limit of our study is that we did not measure the exact flow rates for all of our experiments at 150 and 300 mmHg. This would have been helpful since the literature published by blood warmer manufacturers often cite flow rates. However, even if it was not systematically measured in our experiments, it was observed that we needed about 17 seconds to reach 25 mL of RBC in a graduated cylinder at 150 mmHg, while it was about 10 seconds at 300 mmHg. These observations allow to calculate proxies of 88 mL/minute for 150 mmHg and 150 mL/minute for 300 mmHg.

Another limit of our study is that we tested only one type of blood warmer, namely the Hotline® HL-90, which uses a countercurrent water bath heat exchange system. This system warms the RBC when it flows through the tube before reaching the patient. Other systems can be used to warm RBC, such as dry heat, infrared, microwave, convective air, countercurrent metal [28–29]. The most used are systems using countercurrent water bath, countercurrent metal and dry heat. These three systems gradually warm RBC in the tube, which allow providing warm RBC closer to the patient and avoid excessive heat loss (i.e. the distance to reach by the RBC between the warming device and the patient is shorter as compared to directly warm the whole RBC unit before to transfuse it through the tube). Considering the different available blood warming devices on the market and the distance to travel by warmed RBC before to reach the patient, our results may have been different as regard to temperature at the outlet of the tube if we had used another blood warmer in our experiment. However, we do not think that hemolysis results would have changed since other blood warmers would have been set at the same temperature.

## Conclusion

During massive transfusion protocols the use of a blood warmer adjusted to 41.5°C with a compression sleeve set at 150 or 300 mmHg does not generate hemolysis. However, at a pressure rate of 300 mmHg, the temperature of RBCS transfused to the patient does not avoid a risk of hypothermia with a median temperature of 33.7°C. Further investigation should thus be done at higher warming temperature.

## Supporting Information

S1 Table Hemolysis with blood warmer at 41.5°C and compression sleeve at 150 and 300 mmHg. (DOC)

S2 Table Temperature at the exit of the blood warmer. (DOCX)

## Abbreviations

RBC: red blood cell
mmHg: millimeter of mercury
°C: degree Celsius
mL: millimeter
FHb: free hemoglobin
Hb: hemoglobin
Htc: hematocrit
Rpm: revolutions per minutes
CP2D: double-citrate phosphate dextrose
CBC: complete blood count
HL: Hotline®
