# Supplementary material for: Pressure Infusion Cuff and Blood Warmer during Massive Transfusion: An Experimental Study About Hemolysis and Hypothermia
Source: PLoS One. 2016 Oct 6;11(10):e0163429. doi: 10.1371/journal.pone.0163429 (PMC5053533; doi:10.1371/journal.pone.0163429)
Supplement: S1 Table — (DOC) [file pone.0163429.s001.doc]

**S1 Table. Hemolysis with blood warmer at 41.5°C and compression sleeve at 150 and 300 mmHg**

|  | Hemolysis (%) | | | Free hemoglobin (g/dl) | | |
| --- | --- | --- | --- | --- | --- | --- |
|  | 150  mm Hg | 300  mm Hg | P-value | 150  mm Hg | 300  mm Hg | P-value |
| *Transfusion with a compression sleeve* | 0,26 | 0,19 | 1 | 0,08 | 0,07 | 0,98 |

Note : values are for the median.
