# Supplementary material for: Pressure Infusion Cuff and Blood Warmer during Massive Transfusion: An Experimental Study About Hemolysis and Hypothermia
Source: PLoS One. 2016 Oct 6;11(10):e0163429. doi: 10.1371/journal.pone.0163429 (PMC5053533; doi:10.1371/journal.pone.0163429)
Supplement: S2 Table — (DOCX) [file pone.0163429.s002.docx]

**S2 Table. Temperature at the exit of the blood warmer**

|  | Temperature with the blood warmer set at 41,5°C  (median)  (min, max) |
| --- | --- |
| *Compression sleeve at 150 mm Hg* | 37,10  35,70; 38,60 |
| *Compression sleeve at 300 mm Hg* | 33,65  32,60; 34,50 |

Note: A temperature measured in accordance with the expected temperature must have a value within the theoretical range [41.0-42.0].
